# The Use of Immersive Environments for the Early Detection and Treatment of Neuropsychiatric Disorders

**DOI:** 10.3389/fdgth.2020.576076

**Published:** 2021-02-04

**Authors:** Robert F. K. Martin, Patrick Leppink-Shands, Matthew Tlachac, Megan DuBois, Christine Conelea, Suma Jacob, Vassilios Morellas, Theodore Morris, Nikolaos Papanikolopoulos

**Affiliations:** ^1^Department of Computer Science and Engineering, University of Minnesota, Minneapolis, MN, United States; ^2^Department of Psychiatry and Behavioral Sciences, University of Minnesota, Minneapolis, MN, United States

**Keywords:** tourette, autism, behavioral disorder, neuropsychiatric, immersive environment

## Abstract

Neuropsychiatric disorders are highly prevalent conditions with significant individual, societal, and economic impacts. A major challenge in the diagnosis and treatment of these conditions is the lack of sensitive, reliable, objective, quantitative tools to inform diagnosis, and measure symptom severity. Currently available assays rely on self-reports and clinician observations, leading to subjective analysis. As a step toward creating quantitative assays of neuropsychiatric symptoms, we propose an immersive environment to track behaviors relevant to neuropsychiatric symptomatology and to systematically study the effect of environmental contexts on certain behaviors. Moreover, the overarching theme leads to connected tele-psychiatry which can provide effective assessment.

## 1. Introduction

Neuropsychiatric disorders, especially those which begin in childhood, are highly prevalent conditions that often remain chronic without effective treatment, which can lead to persistent disability and impairment across the lifespan. Recent research suggests that about 15% of youth are diagnosed with a mental disorder before 18 years of age (1). The ability to detect the onset of these conditions at the earliest possible time is critical for improved heath outcomes later in life. Critically, effective care hinges on accurate analysis of symptom presence and severity: clinicians must measure symptoms precisely in order to reach the proper diagnosis, identify an appropriately targeted treatment plan, and track the benefit of interventions. However, current methods for neuropsychiatric symptom assessment are severely limited.

Existing tools are limited to paper and pencil self-report measures of symptom severity and impairment, questionnaires, clinician-administered interviews, and observations (2, 3) that are prone to numerous rater biases and entail significant training burden for a clinician to achieve reliability. Reliance on observations can lead to differing results based on the context of those observations [e.g., parent vs. clinician or school vs. home; (4)]. These limitations can have detrimental consequences, including misdiagnosis of children, leading to unnecessary medical or psychiatric treatments (5), or under-diagnosis, such that children do not receive necessary mental health interventions. It is therefore critical to develop sensitive, quantitative tools to accurately identify early subtle neuropsychiatric abnormalities, improve diagnostic precision, and inform objective monitoring of treatment effects.

Many neuropsychiatric disorders display observable behavioral abnormalities that could be used to inform a quantitative evaluation. For example, motor behaviors are core symptoms in several neurodevelopmental disorders, such as motor and vocal tics in Tourette Syndrome (TS), restricted-repetitive behaviors in autism, compulsions in obsessive-compulsive disorder (OCD), and hyperactivity and motor overflow behaviors in attention-deficit hyperactivity disorder (ADHD).

Discussions in medical practice today focus on the value of early detection and intervention since they are often connected to better patient outcomes. Thus, the field of mental health is putting major emphasis on early detection (6). However, neuropsychiatric disorders typically begin with very subtle symptoms or prodromal indicators that can go missed by existing assays. The result is that detection and intervention may come too late in the disease process. Thus, tools that enable screening for the earliest manifestations of neuropsychiatric illness may help improve our ability to alter the illness trajectory by delivering prevention or intervention as close to disorder onset as possible. The major challenge is to discover quantifiable signs of neuropsychiatric illness (e.g., behaviors, neuromotor differences, physiological responses) that enable intervention at the first opportunity. Finally, clinicians want to accomplish these objectives in a connected health setting which enables effective assessments no matter where the patient or clinician are located.

## 2. Background

### 2.1. Existing Tools to Assess Neuropsychiatric Disorders

Significant research in recent years has sought to develop comprehensive and reliable assessments to measure the presence and severity of symptoms of neuropsychiatric disorders. Current best-practices generally include administering batteries of multi-informant assessment measures comprised of psychometrically validated parent, teacher, and self-reported questionnaires and structured, clinician-administered rating scales. Parent-report measures typically include questions about observed child behavior and are intended to provide information about the extent to which symptoms exist and interfere with daily life. Clinician-rated scales typically involve a clinician making ratings of symptoms and their functional impact based on observation and information obtained via structured or semi-structured interview. Reports for these measures assume accurate reporting from parents, caregivers, and clinicians, but their subjective nature means that results can be influenced by rater biases [i.e., variability among raters due to differential interpretations of the question/rating scale or unique/divergent perceptions of the topic or individual being rated; (7)], response biases (e.g., social response biases, demand characteristics, recall/recency effects), or instrument biases (e.g., question ordering; cultural, semantic, or conceptual biases in the design of a questionnaire). Children, especially with disorders that affect communication, may not be able to articulate symptoms fully and will subsequently under-report symptoms. Similarly, parents may be consistently susceptible to under- or over-reporting depending on their understanding of the questionnaire and their desire for treatment (8). In addition, there are known challenges for interpreting these questionnaires. For example, research shows a lack of concordance between and parent and child reports of symptoms and symptom severity in ASD and ADHD (9). High rates of comorbidity among neuropsychiatric disorders further complicates interpretation of assessment measures because symptoms often overlap beyond a single categorical diagnosis or multiple independent diagnoses (10).

Parent-report questionnaires and clinician-administered interviews are intended to inform a categorical diagnosis about the presence or absence of a neuropsychiatric disorder and to assess symptom severity. This is an important first step for intervention access and planning, as well as for understanding how pervasive a disorder might be, but may not always provide specialized information about how to personalize treatment planning. There is a pressing need for measurement techniques that capture quantifiable symptoms objectively.

### 2.2. Early Detection

Although the onset and course of symptoms can differ between specific neuropsychiatric disorders, symptoms of some conditions can emerge as early as infancy and progress throughout development. Symptoms can also emerge during various developmental windows where risk for certain disorders is heightened, such as adolescence and the transition to adulthood. These developmental windows are characterized by periods of high neuroplasticity in which certain individuals are especially susceptible to developing symptoms of psychiatric disorders (11). Screening during these particular times in development can lead to earlier detection, which can ultimately help attenuate symptoms and slow the progression of a disorder.

In disorders that emerge during critical developmental periods, as well as in disorders that emerge in infancy, the delay between onset and diagnosis is significant, and treatment often does not begin until years later (12). Detecting symptoms at an early age is crucial for an earlier and more accurate diagnosis, and is crucial for improving outcomes long-term. For example, research has shown that detecting schizophrenia early, before debilitating symptoms emerge, can slow the course of the disorder and significantly improve outcomes (13, 14). Furthermore, because there are high rates of comorbidity in neuropsychiatric disorders, early intervention may curb effects of other related disorders that emerge later in the course of a disorder and worsen both physical and mental health.

### 2.3. Technological Approaches

Technology has many times been created for and applied to performing labor-intensive and tedious tasks. In the past century, advancements in computer technology have solved problems that typically involved intense computation or inordinate/vast amounts of data. However, with the low cost of cameras and increasing computational power of computers problems involving observation or surveillance are now being addressed. Neuropsychiatric disorders, such as TS, OCD, and autism usually require a close visual assessment by a trained clinician as a component of the diagnosis. As the time of clinicians is a scarce resource, this is an area ripe for exploration.

As behavior disorders involve motion, there have been a number of different approaches to analysis. Bernabei et al. (15) used wearable accelerometers to track the body movement of a number of individuals with TS. Participants were visually recorded standing still and walking for a short period of time. The videos were annotated by trained clinicians and the acceleration data were analyzed for motion spikes. The researchers found good agreement between automatic and clinician tic detection but found that the method was limited in the range of tics that could be captured. One approach to Parkinson's disease (PD), a nervous system disorder that affects movement, was to insert pressure sensors into the soles of shoes (16). Using stride data gathered from the sensors, a hidden Markov model (HMM) was trained and used to discriminate between healthy subjects and those suffering from PD. Acoustic signals are another sensing modality that have been applied (17), along with a variety of machine learning techniques, to diagnose patients with PD with good results. Using recordings of speech patterns researchers were able to classify patients suffering from PD with an accuracy rate of larger than 95%.

Various studies have also used images and videos of a participant's face to detect psychiatric disorders. The human face not only expresses emotion but its motion also indicates an individual's mood. Scherer et al. (18) found four distinct descriptors using the eyes and lips to determine a correlation with symptoms of depression, anxiety, and post-traumatic stress disorder (PTSD). A wider range of emotions and mental states were recognized in Grafsgaard et al. (19) as predictor of educational outcomes. Using smaller features like the eyebrows, eyelids, or chin, researchers were able to determine when a participant was engaged in learning and when they became frustrated.

Autism spectrum disorder (ASD) is another neuropsychiatric disorder where technology can be applied to great effect. ASD is typically diagnosed through close observation during a clinic visit, which requires focused attention on the part of the clinician and may not be the most comfortable setting for the patient. A recent survey (20) focused on the applications of mobile computing for ASD early detection and remote monitoring and concluded that these efforts “will have significant clinical impact.” Using multiple cameras, a child and parent were recorded doing structured play in a natural setting in Rehg et al. (21). Various behaviors were detected, such as gaze direction, smile detection, and object engagement, and these behaviors can be used as metrics to assist in the diagnosis of ASD. A recent study (22) has used an overhead camera and a small humanoid robot to detect and quantify interactions between a child and the robot. These can then be used to help diagnose and treat those with ASD. Other research groups (23, 24) used visual features like the Histogram of Gradients (HOG), the Histogram of Optical Flow (HOF), or SURF features to facilitate the analysis.

Video has also been used to analyze OCD. In Zor et al. (25), participants were recorded performing specific actions and the video was manually annotated. Using this annotation, researchers were able to discriminate between two types of compulsions: cleaning and checking. While the video was not automatically analyzed, a main conclusion from this research was that video analysis “offers an objective and practicable method by which to facilitate discernment” of OCD. Kim et al. (26) used a mixed group of participants with and without OCD to navigate through a virtual reality (VR) world to perform a variety of tasks. Using their interaction data with this virtual world researchers were able to differentiate between the two groups and further tests showed that checking behaviors could be elicited in virtual environments.

### 2.4. Immersive Environments

Immersive environments allow a person to interact with a computer-generated world. There are varying degrees of immersion based on which senses (e.g., sight or hearing) are simulated and to what degree a participant can control the world. Simple setups include head-mounted displays that allow 360° viewing while more complex uses include display, headphones, and haptic feedback to navigate the world. There are obvious applications in the domains of entertainment, education, and architecture, but in the last decade virtual reality has also been applied to medical diagnostics and treatments.

The ability to control an environment to induce stimuli has shown promise for general assessment (27) as well as possible treatments for specific disorder (28). Recent work (29) has shown that immersive environments, tailored to an individual's fear stimulus, can be used along with cognitive behavioral therapy (CBT) for children with ASD. In general, immersive environments have shown great potential benefit in this area (30). Recent research has looked at the applicability of VR toward the study of behavioral disorders. Oagaz et al. (31) created a virtual environment where the participants were asked to perform a task that tested cognitive ability, memory, balance, and motor skills. This study showed the validity of such a system toward the study of neuropsychiatric disorders.

## 3. Instrumentation and Methods

### 3.1. Components

**Figure 1:**
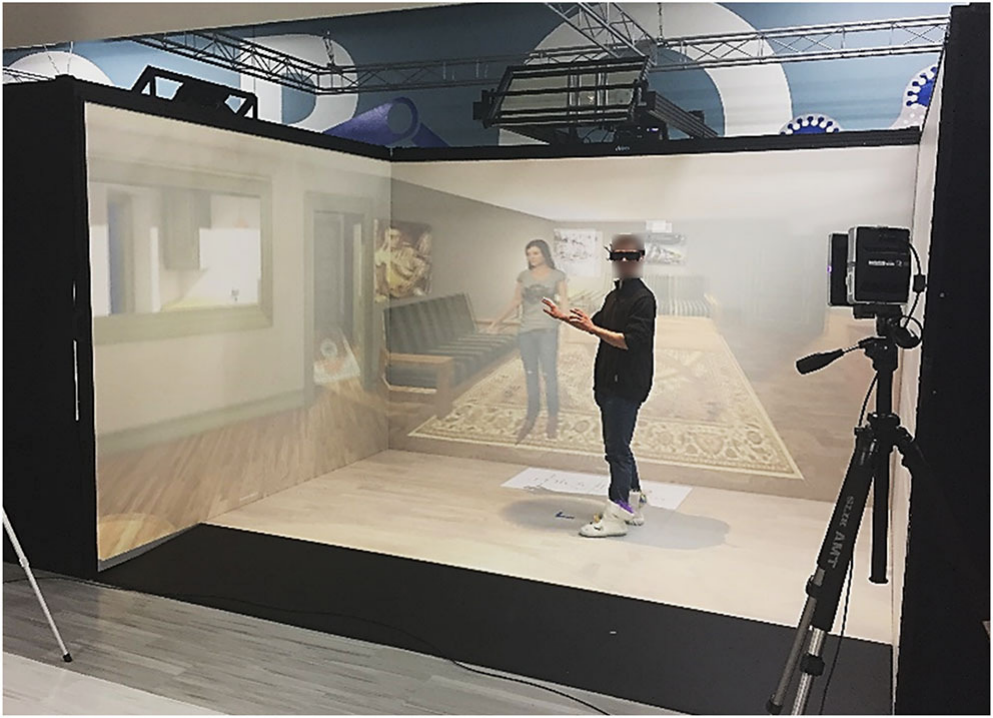
A co-author interacting with the CAVE system.

Robustly sensing and recording movement for detecting behaviors such as tics, compulsions, and restrictive-repetitive behaviors will require a wide variety of different types of data. To this end, several sensors have been added or planned for inclusion. The challenge of synchronizing multi-modal sensor readouts was a major consideration when selecting the specific sensors and sensor types. A typical setup with a subject can be seen in Figure 1.

#### 3.1.1. CAVE

The basis of the system is the Cave Automatic Virtual Environment (CAVE) (32). It is a virtual reality system that projects the user's surrounding onto a set of walls around them. This project uses a VisCube C4, which projects onto three walls and the floor. Unlike more widely available head-mounted virtual reality systems the CAVE allows users to see their own body rather than a rendered replacement. This feature provides an increased level of immersion, though this is lessened by the fact that a user will see outside of the virtual environment if they turn around or look up. Like traditional VR there is a hard limit on the area that a user can traverse and an external controller is necessary if the environment calls for a user to traverse a significant distance.

#### 3.1.2. VICON System

The VICON system provides Vantage infrared cameras and a software tracker to locate objects affixed with retroreflective markers. Four infrared cameras are mounted in the top four corners of the CAVE. It allows for simultaneous tracking of objects which will be very useful during object manipulation tasks. It also includes functionality to record position and orientation information which can be played back in the tracker or exported. Recording can be triggered from the application GUI or via a UDP message. The software package utilizes a Virtual Reality Peripheral Network (VRPN) which relays real-time tracking information from the cameras.

#### 3.1.3. Stereo Glasses

While in the CAVE, users wear Volfoni Edge glasses. These active glasses synchronize with the projectors and allow for the perception of 3D objects, both past the projector walls and inside the area where the user can move. The glasses also include a passive retroreflective tracker array which is tracked by the infrared cameras. The tracking determines the position and orientation of the user's head at all times. This is critical to the functioning of the CAVE as it determines what should be displayed on each projector as the user walks and looks around.

#### 3.1.4. Video Camera Array

Four high-FOV Mobius Maxi cameras are mounted overlooking the CAVE. While the VICON cameras are useful for tracking objects, they are unable to record normal video. The Mobius cameras have a setting which allows them to auto-record which is particularly useful for synchronizing data. Each camera can be triggered to record simultaneously via a serial message to a typical micro-controller board such as an Arduino, which in turn triggers a relay module to the cameras.

#### 3.1.5. High Fidelity Microphone

An Earthworks QTC40 omnidirectional condenser microphone is mounted hanging above the CAVE. It was selected because it is designed to detect very quiet sounds and capture a true representation of high and low frequencies. It is connected to an Apollo Twin USB audio interface which interfaces with the computer that runs the CAVE. In combination with the other sensors it can be used to help try to identify correlations between the subjects environment and the expression of behavioral anomalies, such as vocal tics.

#### 3.1.6. Xsens Human Body Tracking System

The VICON and Mobius cameras both are susceptible to subject and object occlusion. To complement the cameras, an Xsens MVN Awinda will help track body position even when views are occluded. The system includes a vest and straps to attach 17 wireless inertial measurement units (IMUs) to major joints on a user's body. The accompanying software, MVN Analyse, fuses the IMU data, and provides a robust pose. UDP messaging is used to start and stop recording pose information making it easy to synchronize with the other data.

### 3.2. Integration

Because the projection onto the walls of the CAVE is informed by the head tracking of the user from the VICON cameras, a communication protocol is needed to transfer information from the tracker. This protocol should also work between separate machines, as it can be desirable to run the tracker and the CAVE on different computers for the sake of computational efficiency or as a result of the physical location of hardware. The VRPN is designed for this task. It allows for simple communication of tracking information, as well as any other desired peripherals such as game controllers, across a system.

**Figure 2:**
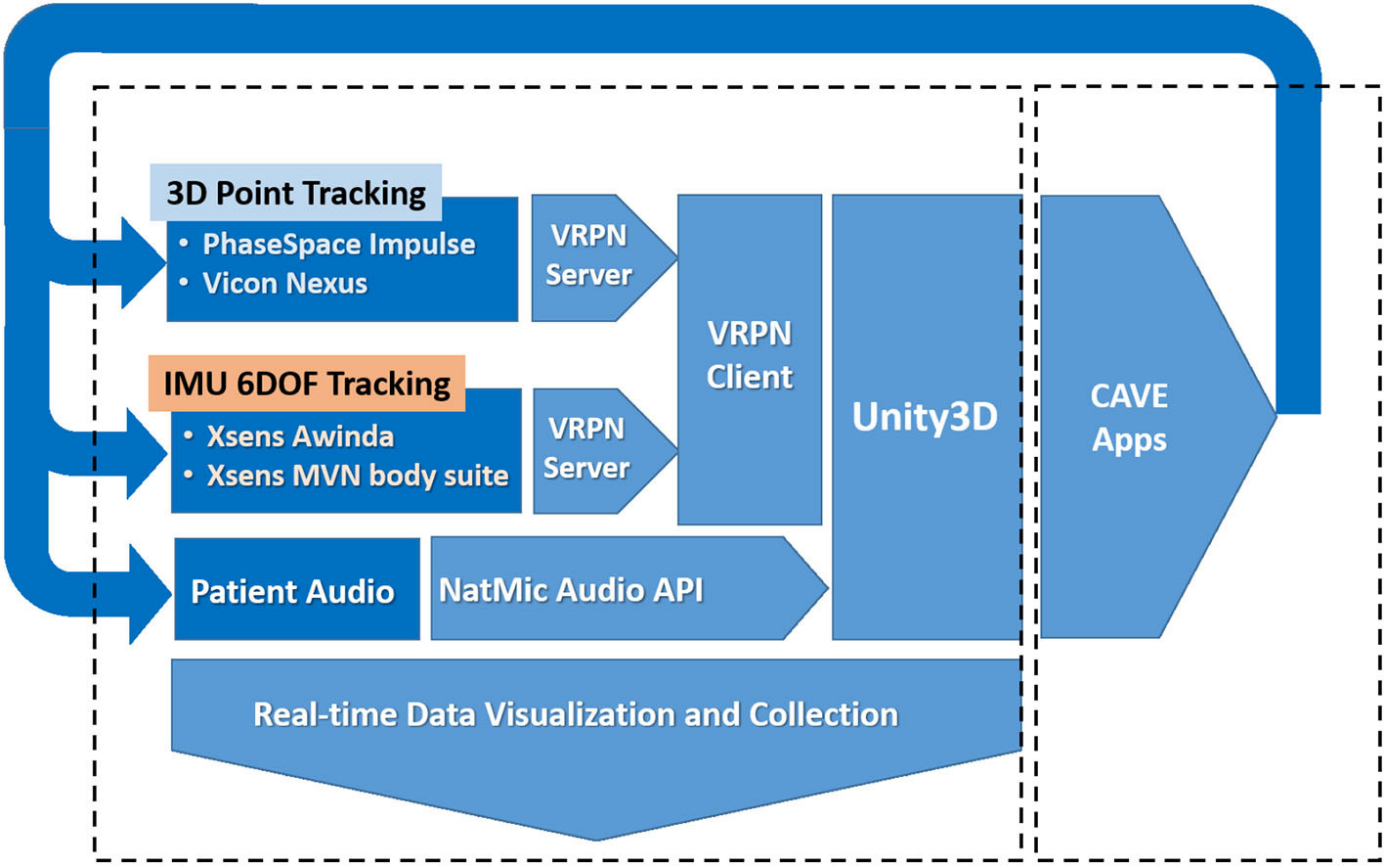
Diagram of the system components.

The software MiddleVR is used to describe the physical projected display layout and to then update the perspective Left/Right eye view transformations used to render the world on each of the 4 display surfaces (33), using the acquired 3D position and orientation of the subjects head through the VRPN. MiddleVR also interfaces directly with other peripheral devices that can used by applications for testing and development (game pads, 3D wands, Mouse, Keyboards, and so on). The software also coordinates the 3D rendering pipeline to the projected 2D displays represented within the Windows graphics device driver. The representation appears as a second monitor display comprised of the four 2D display surfaces joined vertically together into a single tall display. The system architecture is shown in more detail in Figure 2.

MiddleVR also provides a plugin for the Unity3D game engine to aid in world creation for the CAVE. This allows for any information from VRPN and any other devices monitored by MiddleVR described previously to be read directly in Unity3D. The VICON tracking data is particularly useful as it allows a simulation to react to the user's position and the motion of any custom controllers with retroreflective arrays attached to them. The plugin also allows the world to be compiled in such a way that it can be properly displayed on the CAVE.

#### 3.2.1. Synchronization

With many sources of data being recorded, synchronizing the data collection is vital. While it would theoretically be possible to read live streams of data from every sensor and then collect and save all the available data at a given time, this doesn't leverage the built-in recording functions of the various programs and devices and would likely result in reduced frequency of data sampling. The simplest solution is to ensure that each device is triggered to begin recording at the same time. This way all of the data being collected is synchronized without the need to record and later adjust for global timestamps.

In addition to world building, Unity3D is also used for synchronized triggering of recording. Attaching a C# script to an object in a Unity3D scene exposes functions that run at the creation and destruction of the object, which translate to the beginning and end of an application if the script is attached to an object that is always present in the scene. These methods are used to send UDP messages to Tracker and Analyze, to send serial messages to the microcontroller attached to the Mobius cameras, and to run commands to start and stop the microphone recording. This achieves the desired data synchronization between the independent recording methods.

## 4. Results

### 4.1. Preliminary Results

**Figure 3:**
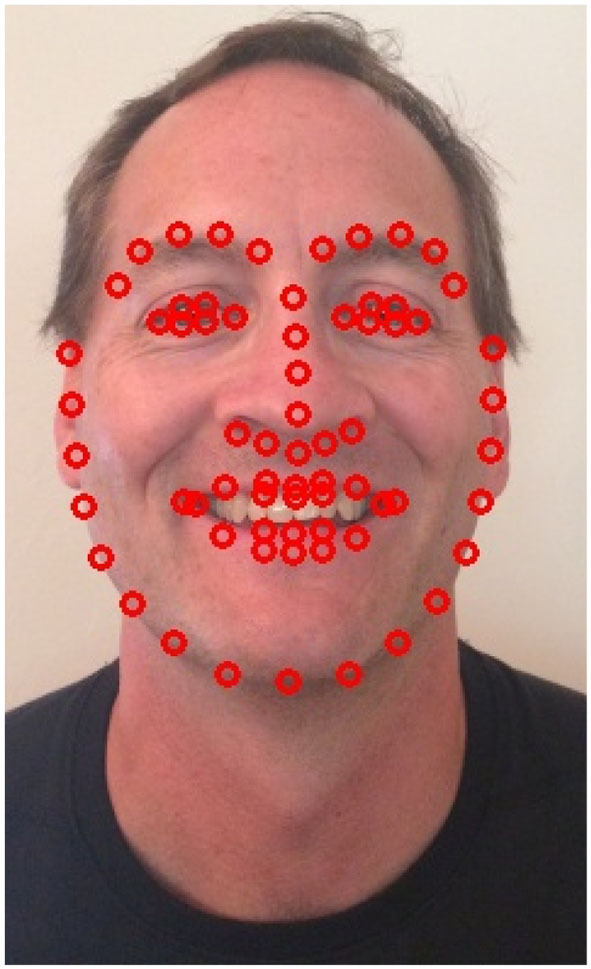
Facial landmarks detected on a co-author.

**Figure 4:**
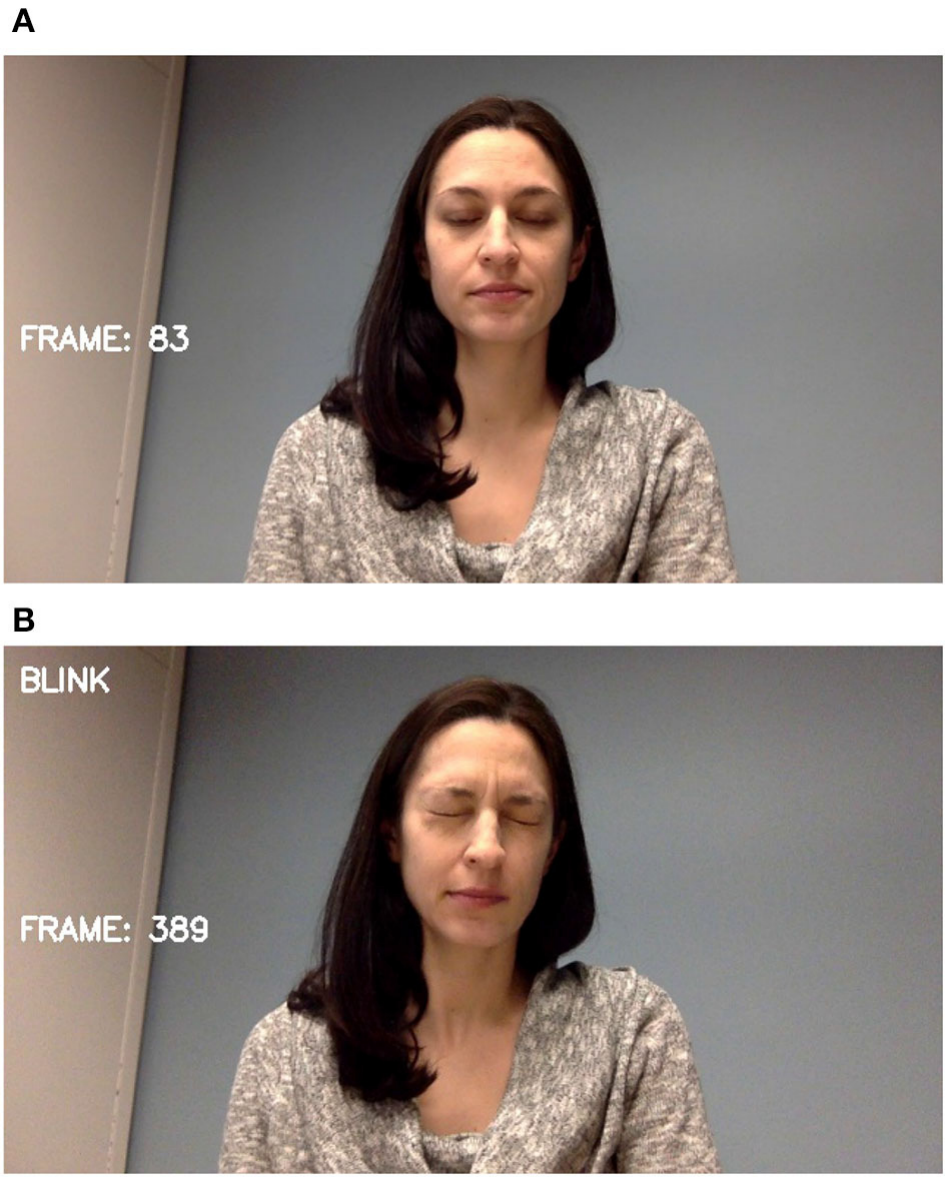
Stills from video of co-author: **(A)** blinking normally, **(B)** mimicking tic behavior.

The two most prevalent tic behaviors involve the eyes and the mouth. Close observation of the face allows for the detection of nearly half of typically expressed tics (34). Facial landmarks are detected and tracked as shown in Figure 3. For blinks, the relative locations and shapes of the eyes and eyebrows are computed and linear discriminants are used to distinguish blinking behavior as seen in Figure 4. Ongoing work is investigating the use of the complete set of facial landmarks coupled with a recurrent neural network (RNN) to further separate tic-like blinking from normal blinking.

Subjects with OCD show quantifiably different behaviors compared to a controlled population on certain common daily tasks. Experiments were designed to invoke and quantify behavioral markers associated with OCD by performing common tasks within mock-up environment scenarios. We recorded video of subjects with OCD and a control group performing free-arrangement tasks and hand-washing tasks (35). A bathroom environment was constructed for subjects to perform hand-washing tasks. A soap dispenser, and drying towels were initially positioned next to a sink/faucet and toilet. The video cameras recorded top-down/frontal perspectives of 39 subjects performing the instructed tasks. Eighteen children were diagnosed with OCD, mean age 11.5 years, on the Yale-Brown Obsessive Compulsive Scale (CY-BOCS), with 21 children, mean age 10.7 years, representing the control group. Additional clinical assessment scales were completed by the child and parents. Parents completed the Child Obsessive-Compulsive Impact Scale-Revised (COIS-R) and Behavioral Assessment System for Children, Second Edition (BASC-2). The children also completed the COIS-R as well as the Multidimensional Anxiety Scale for Children-2 (MASC-2). For repetitive behavior quantification, revisiting similar objects and locations were manually counted and denoted in time by several blinded raters to address subjective bias. The total time to finish the tasks was also encoded by the raters.

Compared to the control group, OCD subjects were significantly more likely to exhibit other “extraneous” behaviors such as, the repetitive behaviors of touching/tapping, washing and drying the sink, and extensive exploration of the space (*p* < 0.003). The time duration to complete hand-washing task was also positively correlated with their CY-BOCS scores (*R* = 0.54, *p* < 0.05) and as well as with the CY-BOCS ordering/repeating dimension (*R* = 0.57, *p* < 0.05). The hand washing duration time indicated similar statistical correlation significance with the other administered test scores as well. Although the individual extraneous behaviors may be a useful behavioral marker, they were instead aggregated into a single measure due to limitations in the manual observation process to extract and quantify more detailed motion characteristics. Accordingly, we also examined the discriminative efficacy of several motion-based feature descriptors, derived from the automated extraction of the hand image-based motion trajectories (dense Farnsworth optical flow estimation) from the recorded video data (36). Generally, features based on the image pixel level trajectories of the subject hands, or their estimated velocities, correctly categorized over 80% of the subjects between the two groups. The results are considered preliminary due to the small sample size. In addition to testing more subjects to validate these findings, accurate motion trajectory information will be collected concurrently with the video to corroborate human observations and extract more complete behavioral marker characterizations. It has also been hypothesized that environment is a factor in compulsion expression. Our investigations will also test the effects of changing spatial layout, sound, and illumination on eliciting these behavioral marker differences in order to practice suppression techniques.

### 4.2. Anticipated Results

One anticipated result is the ability to consistently assess study participants or clinical patients at different times and locations. A difficulty in many current treatment regimens is the lack of time and resources to expertly quantify clinically relevant behaviors before, during, and after treatment. In this case, the data gathered with this instrument can be analyzed at any time and compared to other similar observations. There is also potential to utilize the instrument itself to deliver intervention. For example, a number of studies have consistently demonstrated (37) that children are better able to control tics when rewards are given for suppression. This system could “gamify” tic suppression through automatic tic detection and environment manipulation, opening up the possibility for standalone, game-like programs to train and support the ability to voluntarily suppress tics or for using such a tool to augment existing behavioral interventions that aim to improve tic control via skills training.

Additionally, there has been recent work that shows a correlation between galvanic skin response (GSR) and tic expression and frequency (38). One application of this instrument would be to connect the GSR with other external sensations (i.e., auditory or visual) to investigate strategies for tic suppression using biofeedback. GSR alone was not shown to create statistically significant reduction in tic frequency (39) but this shows the need for further research.

### 4.3. Use of Instrument

With the number of diverse components of the system it is important that the instrument be as streamlined as possible. A researcher needs to simultaneously power on the CAVE projectors, VICON cameras, audio interface, and the micro-controller used to power and synchronously start Mobius camera recording. Subsequently, the researcher then needs to launch the tracker software and verify that the necessary VICON cameras are online. Following this there is a currently time-intensive calibration step for the Xsens tracking suit. Finally, any custom Unity3D applications can be started. At this point, the system is ready to be run.

## 5. Conclusion

The proposed system has broader impact than the domain of neuropsychiatric disorders. Education will be one of the primary beneficiaries of this system. Researchers in areas from computational complexity to computer vision to immersive environments will be able to design new experiments and collaborate with colleagues and students from varied backgrounds. Cross-disciplinary research encourages new ways of thinking about a problem and enhances critical thinking skills for all involved.

One of the most unique aspects of the proposed system is the ability to generate real-time, integrated feedback. The CAVE allows a participant to see the context, trigger, and resulting behavior as it occurs. This allows for more efficient symptom monitoring and training. Specific scenarios can be recreated and replayed at will until desired results are achieved.

Additionally, this immersive environment has the capability to make objective, quantifiable measurements. Most assays currently require a specifically trained human observer who nonetheless introduces bias and subjectivity. The precise digital recording feature of this system will increase consistency and objectivity of diagnostic. And quite often, human observers may not even be available for remote or rural populations (40, 41). This system and subsequent tools will be able to take the place of specialists where none are available or are not easily accessible (digital connected health). Because of the digitization process of the data, results can be easily sent for analysis to any location. This will greatly expand current treatment and diagnostic options for large segments of under-served populations.

Mental health assessment continues to make progress as diagnostic tools and treatments are refined and standardized. We hope that this progress is accelerated with the unique mix of capabilities of this system. Validated results from this tool will be used to create simpler and more streamlined new tools which can be deployed to other research partners, clinics, and perhaps even homes. With the ubiquity of personal computers and integrated cameras, there is an expectation that lessons learned with the CAVE setup can be utilized by anyone anywhere.

## Data Availability Statement

The datasets presented in this article are not readily available because, the dataset distribution requires permission by the UMN Human Subjects Committee. Requests to access the datasets should be directed to Nikolaos Papanikolopoulos, papan001@umn.edu.

## Ethics Statement

The studies involving human participants were reviewed and approved by IRB UMN. Written informed consent to participate in this study was provided by the participants' legal guardian/next of kin.

## Author Contributions

RM has contributed 20% of the paper content, while the other authors have equal contributions. All authors contributed to the article and approved the submitted version.

## Conflict of Interest

The authors declare that the research was conducted in the absence of any commercial or financial relationships that could be construed as a potential conflict of interest.
